# A radiomic model to classify response to neoadjuvant chemotherapy in breast cancer

**DOI:** 10.1186/s12880-022-00956-6

**Published:** 2022-12-23

**Authors:** Peter McAnena, Brian M. Moloney, Robert Browne, Niamh O’Halloran, Leon Walsh, Sinead Walsh, Declan Sheppard, Karl J. Sweeney, Michael J. Kerin, Aoife J. Lowery

**Affiliations:** 1 Department of Surgery, Clinical Sciences Institute, University Hospital Galway, Galway, Ireland; 2 Department of Radiology, University Hospital Galway, Galway, Ireland; 3 Discipline of Surgery, Lambe Institute for Translational Research, National University of Ireland, Galway, Ireland

**Keywords:** Breast cancer, Radiomics, Neoadjuvant chemotherapy, MRI, Biomarker

## Abstract

**Background:**

Medical image analysis has evolved to facilitate the development of methods for high-throughput extraction of quantitative features that can potentially contribute to the diagnostic and treatment paradigm of cancer. There is a need for further improvement in the accuracy of predictive markers of response to neo-adjuvant chemotherapy (NAC). The aim of this study was to develop a radiomic classifier to enhance current approaches to predicting the response to NAC breast cancer.

**Methods:**

Data on patients treated for breast cancer with NAC prior to surgery who had a pre-NAC dynamic contrast enhanced breast MRI were included. Response to NAC was assessed using the Miller–Payne system on the excised tumor. Tumor segmentation was carried out manually under the supervision of a consultant breast radiologist. Features were selected using least absolute shrinkage selection operator regression. A support vector machine learning model was used to classify response to NAC.

**Results:**

74 patients were included. Patients were classified as having a poor response to NAC (reduction in cellularity < 90%, n = 44) and an excellent response (> 90% reduction in cellularity, n = 30). 4 radiomics features (discretized kurtosis, NGDLM contrast, GLZLM_SZE and GLZLM_ZP) were identified as pertinent predictors of response to NAC. A SVM model using these features stratified patients into poor and excellent response groups producing an AUC of 0.75. Addition of estrogen receptor status improved the accuracy of the model with an AUC of 0.811.

**Conclusion:**

This study identified a radiomic classifier incorporating 4 radiomics features to augment subtype based classification of response to NAC in breast cancer.

## Background

Neoadjuvant chemotherapy (NAC), administered prior to tumor resection, plays an established role in the contemporary management of early stage breast cancer. NAC can enable less extensive surgery to be performed and provides valuable prognostic information [1]. NAC generates in-vivo data on tumor chemosensitivity, and response to NAC which is typically assessed by magnetic resonance imaging (MRI) can further inform clinicians of the biological characteristics of the tumor and the patient’s prognosis [2, 3]. Response to NAC is assessed primarily by post-treatment pathology and can be stratified using histologic grading systems such as the Miller–Payne system [4] which classifies response based on the reduction in tumor cellularity. Complete pathological response (pCR) is defined as having no residual carcinoma in the breast tissue following surgery and is associated with an improved prognosis [5].

The pCR rate in breast cancer following NAC ranges from 10 to 50% and is related to patient factors, tumor subtype and NAC regime received [6]. pCR rate is highest among patients with HER2 positive and triple-negative breast cancer (TNBC), while Luminal A breast cancer has the lowest rate of pCR to systemic chemotherapy [7–9]. There is a need for robust and reliable biomarkers to predict response to NAC in order to optimally tailor treatment to individual patients. This could potentially spare a subset of patients, who are unlikely to derive significant benefit from NAC, from the deleterious effects of chemotherapy [10]. Biomarkers in clinical use that improve risk stratification and prediction of response to NAC in breast cancer beyond conventional clinico-pathological factors include Oncotype DX, Mammaprint and ProSigna [11]. These gene-profiling tests require tumor tissue for analysis and while they have utility in recurrence prediction, they were not designed specifically to predict response to NAC. There is a currently unmet clinical need for non-invasive methods to predict response to NAC to further personalize breast cancer management.

Radiomics is a developing field that involves the application of computer-automated software to extract high-throughput quantitative features from radiologic/imaging investigations that can quantify disease phenotype and heterogeneity [12, 13]. Radiomics features include conventional quantitative measurements such as shape/ diameter and surface area. First order features examine the distribution of voxels in isolation and include kurtosis and skewness. Second order features are derived from the spatial relationship between voxels and matrices such as the grey-level co-occurrence matrix (GLCM) [14, 15]. Prediction models using radiomics features incorporating machine learning have shown potential for non-invasive identification of treatment response in breast and other cancers [16–20]. The aim of our study was to develop a radiomics model to predict the response to NAC in breast cancer using pre-NAC breast MRI.

## Methods

### Clinical database

This study was undertaken at Galway University Hospital, a tertiary referral specialist breast cancer unit. This study was conducted in accordance with the granted University College Hospital Galway ethical approval.

Patients who were treated with chemotherapy for breast cancer were identified from a prospectively maintained institutional database including patient demographics, tumor clinicopathology, and surgical and medical therapeutic information. Patients were categorized as receiving NAC or adjuvant chemotherapy based on whether they had treatment before or after their curative surgery. Clinical decisions relating to surgical intervention and neoadjuvant/adjuvant local and systemic therapy are made by discussion and consensus at a multidisciplinary team meeting with medical, surgical, and radiation oncologists present. Residual tumor size and response to NAC were assessed using postoperative pathology of the resected breast specimen by a consultant histopathologist at GUH.

The Miller–Payne grading system is as follows: Grade 1: residual tumor demonstrates no change or some minor alteration in individual malignant cells, but no reduction in overall cellularity. Grade 2: minor loss of tumor cells but overall high cellularity with up to 30% reduction of cellularity. Grade 3: estimated 30% and 90% reduction in tumor cellularity. Grade 4: marked disappearance of more than 90% of tumor cells such that only small clusters or widely dispersed individual cells remain (near pCR). Grade 5: no invasive malignant cells identifiable in sections from the site of the tumor (complete pCR).

**Fig. 1:**
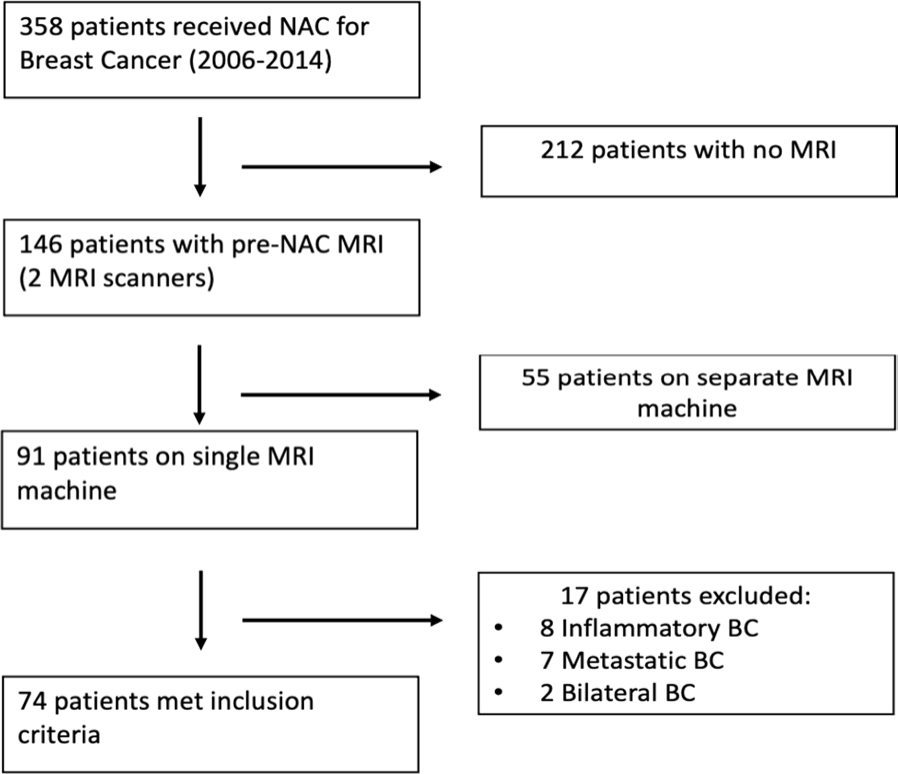
Inclusion criteria flow-chart

Patients with multifocal breast cancer, inflammatory disease and metastatic disease at presentation were excluded from this study (Fig. 1).

### MRI protocol

Magnetic resonance imaging analyses were performed on a short bore 1.5 T magnet (Magnetom Espree 1.5 T, Siemens Healthcare, Erlangen, Germany) using 8-channel breast phase array breast coil for signal reception using the dynamic contrast enhanced (DCE) breast MRI protocol, utilising the following protocol: Sagittal T2 (TR/TE 6570/111, Gap 1 mm, Flip angle 160°, Matrix 340 × 75), Axial T2 FS fl3d pre contrast (TR/TE 5.15/2.39, Gap 0.6 mm, Flip angle 10°, Matrix 320 × 100), Sagittal T1 fl3d (TR/TE 5.18/1.64, Gap 0.6 mm, Flip angle 10°, Matrix 320 × 100, this sequence is repeated 6 times; 1 pre-contrast and 5 post-contrast with peak enhancement in the third run), Axial T1 FS fl3d postcontrast. The section thickness was 3 mm for all sequences. The contrast employed was Gadoterate meglumine (Gd-DOTA). Digitally recreated subtraction image 3-1 postcontrast enhanced was used for final feature analysis.

### Tumor segmentation and radiomic feature analysis

**Fig. 2:**
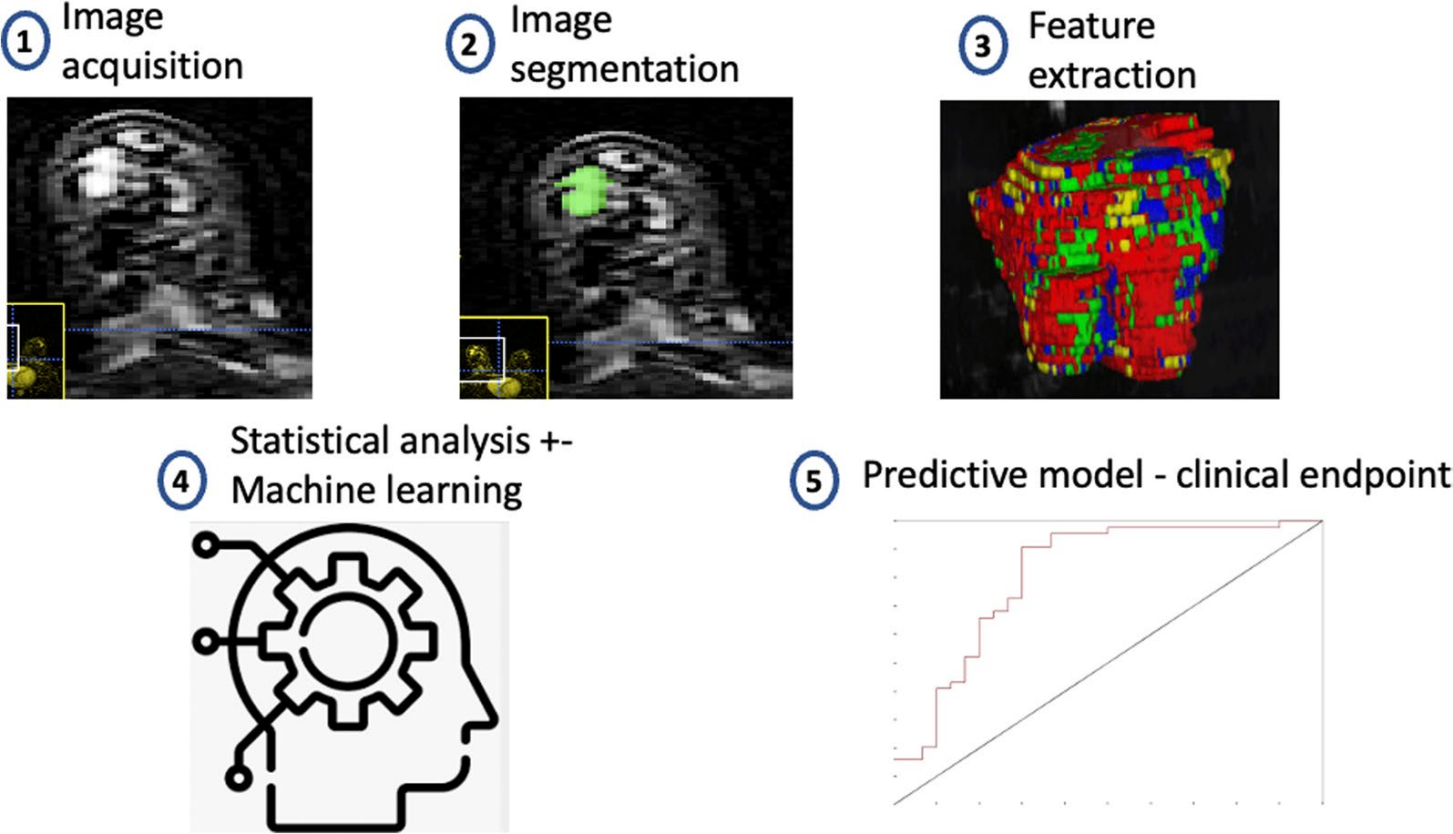
Radiomic pipeline

MR images were evaluated by one researcher (PM) under the supervision of a Consultant specialist breast radiologist (SW). Tumor segmentation was performed by manually delineating the tumor border on axial slices using ITKSnap software (Fig. 2) [21]. Feature extraction was carried out on LIFEx [22], an International Biomarker Standardization Initiative (IBSI)-compliant [23] and validated [24, 25] software package.

An image intensity discretization applying a fixed bin width of 64 was used for feature extraction in MR. Voxel size resampling was performed before feature extraction using cubic interpolation. Images were resampled to isotropic voxels of size 2 × 2 × 2 mm^3^ by 3-dimensional Lagrangian interpolation.

61 features were extracted from each tumor including size and shape features and from several matrices including Histogram-based matrix (HISTO), Grey-level co-occurrence matrix (GLCM), Grey-level run length matrix (GLRLM), Grey-level zone length matrix (GLZLM) and Neighborhood grey-level dependence matrix (NGLDM).

### Radiomic feature selection and machine learning model

To counteract the high throughput nature of data driven radiomics and the high relative correlation between the features produced it is important to first reduce the number of features prior to model training to prevent over-fitting, reduce collinearity and minimize noise [26, 27]. A combination of least absolute shrinkage selection operator (LASSO) regression for feature selection and support vector machine learning (SVM) for model building provide excellent model performance in radiomics and was utilised for this study [28]. LASSO introduces a tuning parameter (λ) that penalizes large coefficients of variables entered into the regression model, reduces the possibility of overfitting [29] and reducing non-pertinent features to zero. In this study, λ was set at 1 and the convergence threshold was 0.0000001. The selected features were used to construct the SVM model with a linear kernel with standardisation pre-processing and a tolerance threshold of 0.001. Receiver operator curve (ROC) was built for the model and area under the curve (AUC) was used to classify model performance.

### Statistical analysis

Data analysis was carried out using IBM SPSS statistics 26.0 and R 4.0.3 with extension XLSTAT v2020.5. A *p*-value of < 0.05 was assumed to represent statistical significance. Continuous variables were summarised using descriptive statistics, including mean, standard deviation, and median. Sensitivity as well as accuracy values were expressed as percentages. Adjustment for confounders was undertaken using multivariable linear or logistic regression for continuous or binary-dependent variables, respectively. The R package “glmnet” was used for LASSO regression and the machine learning module was used to produce the SVM model.

## Results

### Clinicopathological details and response to NAC

Following exclusion of patients with bilateral/ multifocal disease, inflammatory breast cancer and metastatic disease at presentation, 74 patients were included in this study. The majority of patients had invasive ductal carcinoma (n = 56), T2/T3 tumors and luminal A biologic subtype (n = 40) (Table 1).

**Table 1:** Patient clinico-pathological details

	n = 74
Age (mean) +− SD	48.6 (7.9)
Time from NAC- surgery (m)	5.1 (1.1)
*T stage*	
T1	5
T2	35
T3	30
T4	4
*N stage*	
N0	21
N1	39
N2	10
N3	2
N4	2
*Histological subtype*	
Ductal	56
Lobular	10
Mixed	8
*Molecular subtype*	
Luminal A	39
Luminal B	16
Triple negative	15
HER2	4

Response to NAC was assessed using the Miller–Payne response classification based on reduction in tumor cellularity. Response was further stratified into poor (< 90%, n = 44) and excellent (> 90%, n = 30) response to NAC (Table 2).

**Table 2:** Miller–Payne response to NAC

Miller–Payne reponse to NAC	Reduction in cellularity (%)	N = 74
1	< 10	n = 3
2	10–30	n = 6
3	30–90	n = 35
4	> 90	n = 15
5 (pCR)	100	n = 15

Response to NAC differed significantly between subtypes, with triple negative and HER2 disease achieving the highest rates of pCR (Table 3). Patients with luminal A breast cancer (n = 39) were significantly less likely to achieve a pCR compared to non-luminal A patients (n = 35) (5% vs. 37%, *p* < 0.001) and less likely to have an “excellent” response to NAC (> 90% reduction in cellularity), (23% vs. 60%, *p* < 0.001).

**Table 3:** Response to NAC by subtype

Subtype	Poor response to NAC (< 90% reduction in cellularity)	Excellent response to NAC (> 90% reduction in cellularity)	Excellent response	Complete pathological response (pCR)	pCR
Luminal A	n = 30 (77%)	n = 9 (23%)	n = 9 (23%)	n = 2 (5%)	n = 2 (5%)
Luminal B	n = 7 (44%)	n = 9 (56%)	n = 21 (60%) *p* < 0.001	n = 6 (38%)	n = 13 (37%) *p* < 0.001
Triple negative	n = 7 (47%)	n = 8 (53%)		n = 4 (27%)	
HER2	n = 0	n = 4 (100%)		n = 3 (75%)	

### Feature selection

**Fig. 3:**
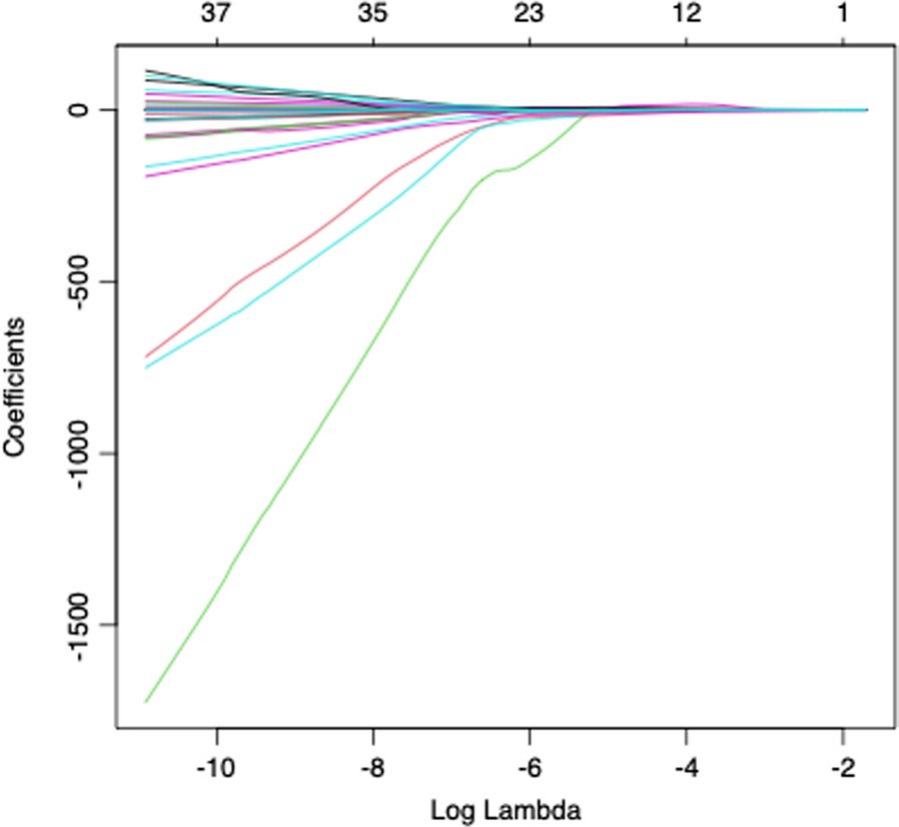
LASSO co-efficients

**Fig. 4:**
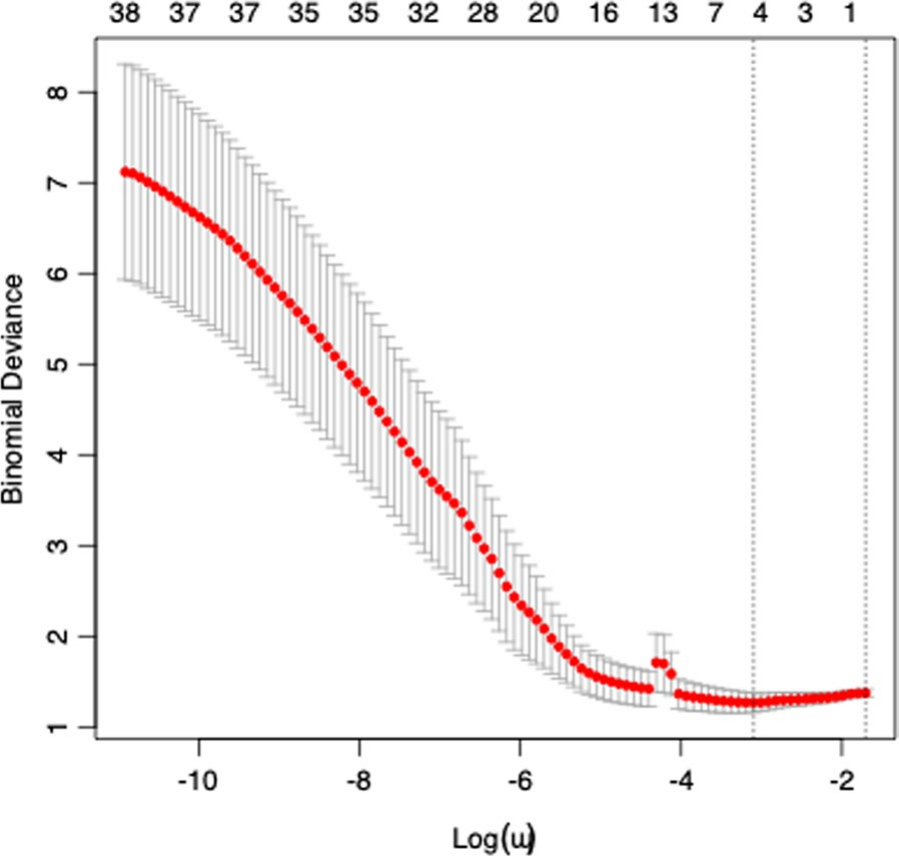
LASSO cross-validation

61 radiomic features were extracted from each tumor. Following LASSO regression, 4 features were selected; (1) Discretized kurtosis, (2) neighbourhood grey-level different matrix (NGLDM) contrast, (3) grey-level zone length matrix short zone grey level emphasis (GLZLM_SZE) and (4) GLZLM zone percentage (ZP) (Figs. 3 and 4).

### Radiomic model

**Fig. 5:**
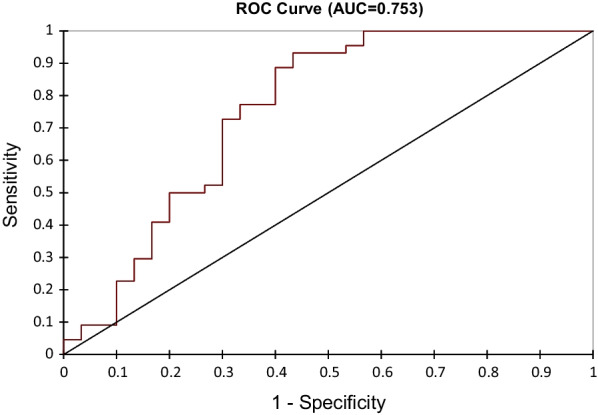
Radiomic model ROC curve

A predictive model was constructed using a SVM approach with the 4 above selected features to differentiate patients with a poor response to NAC (n = 44) from those with an excellent response to NAC (n = 30) (Table 4). This radiomic model demonstrated good predictive performance with an AUC of 0.753 (Fig. 5).

**Table 4:** Radiomic model

	Poor response	Excellent response	Total	%correct
Poor response	38	6	44	86
Excellent response	12	18	30	60
Total	50	24	74	76

### Radiomic model with estrogen receptor status

**Fig. 6:**
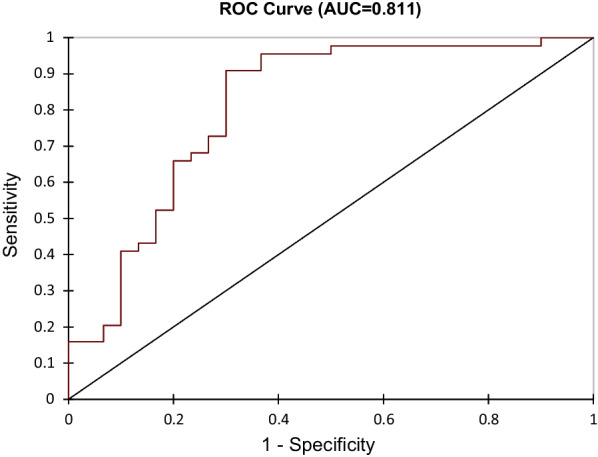
Radiomic model with Estrogen receptor status—ROC curve

Estrogen receptor status was added to the radiomics model, improving the AUC to 0.81. This improved model correctly identified 91% of poor responders and 70% of excellent responders (Table 5, Fig. 6).

**Table 5:** Radiomic model with Estrogen receptor status

	Poor response	Excellent response	Total	%correct
Poor response	40	4	44	91
Excellent response	9	21	30	70
Total	49	25	74	82

## Discussion

NAC is a central element of contemporary breast cancer management, however there is a wide spectrum of response to NAC between patients which can vary based on host, tumor and treatment factors. Patients with locally advanced breast cancer and selected patients with early stage breast cancer are offered NAC with the aim of down-staging the tumor size or gaining valuable information relating to in-vivo tumor response. [30]. Appropriate patient selection for NAC is vital and can be informed by gene expression biomarkers and indexes of proliferation such as ki67. Recent evidence suggests that radiomic features from pre-NAC MRI imaging can be utilised to non-invasively predict response to NAC [31–35] and potentially contribute to the existing patient-selection paradigm.

This study has identified 4 radiomic features from pre-NAC MRI to stratify a cohort of 74 patients with invasive breast cancer into poor and excellent response groups. A machine learning approach was utilised to select these pertinent features and to build a model to predict response to NAC. The addition of estrogen receptor status improved the overall performance of the model, with an AUC of 0.811, identifying poor responders with 90% sensitivity and 70% specificity.

Conventional molecular subtypes help inform likelihood of response to NAC. A study of 838 patients demonstrated significantly different rates of pCR between Luminal A, Luminal B, HER2 over-expressing and Triple-Negative subtypes (6%, 16%, 37% and 38% respectively) [36]. The results of this study compare favourably to similar studies investigating the role of biomarkers in predicting response to NAC. Oncotype DX has been validated in adjuvant therapy, however its’ role in NAC is less clear; a 2019 study of 989 breast cancer patients found that a high Oncotype recurrence score (> 30) was significantly associated with pCR (Odds ratio 4.87) [37]. OncoMasTR is another multi-gene prognostic signature in development specifically to predict NAC response and incorporates 3 master transcription regulator genes as well as tumor size and nodal status [38]. A 2020 study of 813 breast cancer patients showed that OncoMasTR score was significantly associated with pCR (OR 1.68) [39].

Recent evidence supports the addition of radiomic features as potential predictors of NAC response, including in breast cancer [40]. A 2019 study investigating histopathological residual cancer burden in 38 breast cancer patients utilised 23 pharmacokinetic features obtained from DCE-MRI in addition to conventional pathological factors to classify response to NAC with an AUC of 0.92 [34]. A 2020 study of 222 breast cancers utilised a model composed of 12 MRI-derived radiomics features in addition to molecular subtype to identify pCR, producing an AUC of 0.8 using a random forest machine learning approach [31]. In cervical cancer, a 2019 study of 275 patients demonstrated an AUC of 0.999 in predicting response to NAC [41, 42].

The radiomic features identified and tested may further describe the intrinsic tumor environment and the degree of intra-tumor heterogeneity which may impact NAC response. Kurtosis can be used as a measure to assess deviation from the normal distribution of pixel values. Invasiveness may be explained by the degree of pixel-kurtosis in breast cancer [43] and has been shown to be associated with response to chemotherapy in pancreatic cancer [44]. Texture based features evaluating the relationship between pixels are produced by using spatial grey-level dependant matrices. NGLDM (neighbourhood grey-level different matrix) describes the difference in grey-levels between 1 voxel and it’s 26 neighbours in 3 dimensions. NGLDM contrast corresponds to intensity difference between neighbouring regions, and this is the first report of this radiomic feature in association with cancer prognosis or response to chemotherapy.

Grey-level zone length matrix (GLZLM) provide information on the size of homogenous zones for each grey level in 3 dimensions. GLZLM_SZE (short zone emphasis) is a measure of the distribution of the short homogenous zones in an image, while GLZLM_ZP (zone percentage) measures the homogeneity of the homogenous zones. Indices derived from the GLZLM, in addition with kurtosis, were significantly associated with overall survival in a study investigating radiomics features in gastric B-cell lymphoma [44]. A 2020 study that assessed PET scan radiomic features of patients with pancreatic cancer demonstrated that GLZLM non-uniformity was significantly associated with one-year survival and could stratify patients into survival categories [45].

Radiomic features alone show great promise in the stratifying response to NAC, and models incorporating a combination of radiomics and molecular feature are superior [46, 47]. It is conceivable that radiomic features could be a component of future multi-omic panels including genomic and metabolomic markers to aid in the management of breast cancer [48, 49]. We added ER status to moderately improve the overall accuracy of the model to predict response to NAC and produce a greater accuracy in classifying patients into poor and excellent response groups than conventional molecular subtype alone. However, response to NAC can vary significantly even within subtype and is thought to be as result of intra-tumor heterogeneity [50]. Genomic heterogeneity has been shown to impact treatment response and drive resistance to targeted therapies in cancer [51, 52]. Image-based assessment of tumor heterogeneity, incorporating quantitative descriptors of grey-level relationships mentioned above, could potentially reveal aggressive tumor sub-regions for determining prognosis and treatment [53, 54] and be incorporated into the multi-modal decision process of selecting patients for NAC.

This study has a number of limitations. Firstly, it is a single centre retrospective study. While we were able to establish a discrete number of radiomic features to predict response to NAC, a larger sample size is needed to validate the radiomic model. Because radiomics is itself a developing field, there is a paucity of large cohort, prospective studies assessing the clinical utility of radiomic models. Establishing a robust, reproducible radiomics pipeline as is demonstrated in this study, is vital to integrate radiomic biomarkers into clinical practice in the near future [55].

In our study, tumor segmentation was carried out manually by a single researcher, under the supervision of a Consultant Radiologist. Manual segmentation is at present the most reliable method of establishing a region of interest (ROI) for analysis by radiomics software [15]. However, this method can be subject to inter-observer variability. Automatic segmentation by artificial intelligence shows great promise in solving this issue however is some way from being optimised [56].

Pre-processing of images by resampling has been shown to reduce more repeatable and less sensitive to change results [57, 58]. Here, we used a fixed bin width of 64 and carried out voxel resampling to isotropic voxels of size 2 × 2 × 2 mm^3^ by 3-dimensional Lagrangian interpolation, as described in previous studies assessing breast MRI [59, 60]. Fuzzy pre-processing may enable standardised image pre-processing particularly for non-technical experts prior to performing radiomic analysis [61]. Optimal and standardized pre-processing must be established to ensure reproducibility across radiomics studies [62].

In terms of feature extraction software, LIFEx was utilised that produces radiomics features compliant with the International Biomarker Standardization Initiative (IBSI) [63]. This 2020 initiative describes 169 standardized radiomics features that are reproducible across a number of software platforms and can potentially be investigated as clinical biomarkers. As radiomics evolves in the coming years it is likely that the number of software programmes available will increase and it is imperative that rigorous assessment of features continues to ensure reproducibility and reliability of studies.

In conclusion, this study identifies radiomic features that could potentially contribute to the management of patients receiving NAC for breast cancer.

## Data Availability

All data is available from the corresponding author on request.

## Abbreviations

DCE: Dynamic contrast-enhanced
GLCM: Grey-level co-occurrence matrix
GLZLM: Grey-level zone length matrix
GLZLM_SZE: Grey-level zone length matrix short zone emphasis
GLZLM_ZP: Grey-level zone length matrix zone percentage
GUH: Galway University Hospital
LASSO: Least absolute shrinkage and selection operator
MRI: Magnetic resonance imaging
NAC: Neo-adjuvant chemotherapy
NGLDM: Neighbourhood grey-level different matrix
pCR: Complete pathological response
ROC: Receiver operating characteristic
SVM: Support vector machine
